# Mental health and the effects on methylation of stress-related genes in front-line versus other health care professionals during the second wave of COVID-19 pandemic: an Italian pilot study

**DOI:** 10.1007/s00406-022-01472-y

**Published:** 2022-08-24

**Authors:** Silvia Tabano, Lorenzo Tassi, Marta Giulia Cannone, Gloria Brescia, Gabriella Gaudioso, Mariarosa Ferrara, Patrizia Colapietro, Laura Fontana, Monica Rosa Miozzo, Giorgio Alberto Croci, Manuela Seia, Cristina Piuma, Monica Solbiati, Eleonora Tobaldini, Stefano Ferrero, Nicola Montano, Giorgio Costantino, Massimiliano Buoli

**Affiliations:** 1 Department of Pathophysiology and Transplantation, University of Milan, Milan, Italy; 2 Laboratory of Medical Genetics, Fondazione IRCCS Ca’ Granda Ospedale Maggiore Policlinico, Milan, Italy; 3 University of Milan, Milan, Italy; 4 Department of Health Sciences, Milan, Italy; 5 Unit of Medical Genetics, ASST Santi Paolo e Carlo, Milan, Italy; 6 Division of Pathology, Fondazione IRCCS Ca’ Granda Ospedale Maggiore Policlinico, Milan, Italy; 7 Department of Anesthesia and Emergency-Urgency, Fondazione IRCCS Ca’ Granda Ospedale Maggiore Policlinico, Milan, Italy; 8 Department of Clinical Sciences and Community Health, University of Milan, Milan, Italy; 9 Department of Internal Medicine, Fondazione IRCCS Ca’ Granda, Ospedale Maggiore Policlinico, Milan, Italy; 10 Department of Biomedical, Surgical and Dental Sciences, Milan, Italy; 11 Department of Neurosciences and Mental Health, Fondazione IRCCS Ca’ Granda Ospedale Maggiore Policlinico, Via F. Sforza 35, 20122 Milan, Italy

**Keywords:** Health professionals, COVID-19, Mental health, Epigenetics, Stress-related genes

## Abstract

Healthcare workers experienced high degree of stress during COVID-19. Purpose of the present article is to compare mental health (depressive and Post-Traumatic-Stress-Disorders—PTSD—symptoms) and epigenetics aspects (degree of methylation of stress-related genes) in front-line healthcare professionals versus healthcare working in non-COVID-19 wards. Sixty-eight healthcare workers were included in the study: 39 were working in COVID-19 wards (cases) and 29 in non-COVID wards (controls). From all participants, demographic and clinical information were collected by an ad-hoc questionnaire. Depressive and PTSD symptoms were evaluated by the Patient Health Questionnaire-9 (PHQ-9) and the Impact of Event Scale—Revised (IES-R), respectively. Methylation analyses of 9 promoter/regulatory regions of genes known to be implicated in depression/PTSD (*ADCYAP1, BDNF, CRHR1, DRD2, IGF2, LSD1/KDM1A, NR3C1, OXTR, SLC6A4*) were performed on DNA from blood samples by the MassARRAY EpiTYPER platform, with MassCleave settings. Controls showed more frequent lifetime history of anxiety/depression with respect to cases (*χ*^2^ = 5.72, *p* = 0.03). On the contrary, cases versus controls presented higher PHQ-9 (*t* = 2.13, *p* = 0.04), PHQ-9 sleep item (*t* = 2.26, *p* = 0.03), IES-R total (*t* = 2.17, *p* = 0.03), IES-R intrusion (*t* = 2.46, *p* = 0.02), IES-R avoidance (*t* = 1.99, *p* = 0.05) mean total scores. Methylation levels at *CRHR1, DRD2* and *LSD1* genes was significantly higher in cases with respect to controls (*p* < 0.01, *p* = 0.03 and *p* = 0.03, respectively). Frontline health professionals experienced more negative effects on mental health during COVID-19 pandemic than non-frontline healthcare workers. Methylation levels were increased in genes regulating HPA axis (*CRHR1)* and dopamine neurotransmission (*DRD2* and *LSD1*), thus supporting the involvement of these biological processes in depression/PTSD and indicating that methylation of these genes can be modulated by stress conditions, such as working as healthcare front-line during COVID-19 pandemic.

## Introduction

COVID-19 pandemic resulted to be a highly traumatic and stressful event for general population [1]. Different studies reported an increased frequency of mental conditions and insomnia in previously healthy subjects [2] as a result of social isolation, restrictions and fear of contamination [3]. The effects of the pandemic were even more devastating for specific groups of subjects such as individuals with chronic medical conditions (e.g., diabetes or rheumatoid arthritis) [4, 5] or health professionals who had to face directly the sanitary emergency [6].

From the beginning of pandemic, healthcare workers experienced high rates of anxiety, depression and stress [6]. Being front-line, female gender and young age have been identified as the main factors associated with the development of affective symptoms in health professionals [6]. These aspects have been displayed by different studies conducted in various geographical areas including Italy [7], France [8] and Pakistan [9]. Of note, healthcare professionals working in COVID-19 wards were found to be more prone to develop depression, insomnia and Post-Traumatic Stress Disorder (PTSD) symptoms compared to those working in other wards [10, 11].

With regard to biological aspects, COVID-19 and mental disorders share abnormalities in inflammation and Hypothalamic–Pituitary–Adrenal (HPA) axis [12]. A recent study demonstrated that patients with lymphopenia had an increased risk to receive a psychotropic medication during SARS-CoV-2 infection [13]. On the other hand, prolonged stress associated to events such as COVID-19 is likely to modify different biological systems and predispose health care workers to develop psychiatric symptoms [14]. Of note, some authors reported that high levels of stress are associated with the onset of depressive symptoms together with prominent inflammation [15], cortisol dysregulation [16] and modifications in oxytocin-related pathways [17].

A possible link between exposure to stressing factors, such as looking after COVID-19 patients, and psychiatric conditions in front-line healthcare professionals is represented by epigenetic modifications, induced by an adverse environment and leading to changes in gene expression and neural circuit function [18]. Epigenetic modifications can be defined as changes occurring “above” the level of the DNA sequence, thus influencing gene expression without altering DNA sequence [19]. The most widely known and investigated epigenetic mechanism is DNA methylation, consisting in the addition of methyl groups to the cytosine at dinucleotides cytosine–guanine, termed “CpG” dinucleotides, located at DNA regions involved in gene expression regulation.

DNA methylation is a mechanism involved in the regulation of gene expression in the brain, as demonstrated by several studies [20, 21]. Of note, alterations in DNA methylation in the central nervous system may favor the development of neuropsychiatric disorders, such as major depression, schizophrenia or PTSD [22]. Indeed, individuals exposed to chronic stress conditions showed altered methylation levels of genes, such as *BDNF* and *SLC6A4* [23–25]. In addition, early life stress events, such as the in utero exposure to adverse conditions, can result in the setting of altered DNA methylation patterns of genes controlling emotional aspects of behavior, such as *NR3C1, SLC6A4*, and *OXTR* [26]. Interestingly, alterations in methylation levels established during pregnancy could be stably maintained throughout life, and predispose individuals to psychiatric illness, such as major depression [27, 28]. Finally, available literature indicates epigenetic changes in glucocorticoid, serotonergic and neurotrophin signaling in subjects experiencing intense stress and, therefore, vulnerable to suffer from mood disorders [29].

Notably, a number of researches [30–34] demonstrated that alterations in DNA methylation in peripheral cells could reflect those occurring in brain cells, thus proving new opportunities for studying psychiatric disorders by investigating peripheral epigenetic markers of the illness [35, 36]. Purpose of the present article is to compare front-line healthcare professionals versus the other healthcare workers in terms of mental health (depression and PTSD symptoms) and methylation levels of a panel of stress-related genes.

## Methods

### Study participants

Healthcare professionals working at the IRCCS Policlinico Foundation, Milan, Italy were recruited during the second wave of the COVID-19 pandemic in Italy. Health workers were divided into two groups, based on their role in the COVID emergency:

- 39 frontline health workers, directly involved in the care of COVID 19 patients (from emergency or internal medicine departments; hereinafter referred to as "cases");
- 29 healthcare professionals not directly involved in the care of patients affected by COVID-19 (staff from genetics laboratory or pathological anatomy; hereinafter referred to as "controls").

Exclusion criteria were: (1) subjects with current SARS-CoV-2 infection for the direct effects on mental health and epigenetics; (2) healthcare professional who did not work during the second wave; (3) individuals with a recent traumatic event (e.g., death of a relative or a recent diagnosis of a severe medical condition); (4) pregnancy. Cases and controls were age- and gender-matched.

### Clinical assessment

Demographic (age, gender) and clinical (Body Mass Index-BMI, smoking status, number of cigarettes/day, number of coffee cups/day, number of alcohol units/month, presence of hypercholesterolemia, lifetime history of anxiety or depression, presence of a sedentary lifestyle, frequency and duration of physical activity, frequency of using screen-based media, history of COVID-19 infection) were collected by an anamnestic questionnaire**.** Depressive symptoms were assessed by the Patient Health Questionnaire-9 (PHQ-9), a self-reported 9-item scale frequently used to assess depression in contexts other than psychiatry [37]. A cutoff of 10 or higher identifies individuals with clinically significant depressive symptoms [38]. We used the third question on this scale to assess sleep problems (difficulty falling or staying asleep, or oversleeping). Concerning PTSD, symptoms were assessed using the Impact of Event Scale—Revised (IES-R), a self-assessment scale consisting of three subscales (intrusion, avoidance and hyper-excitation) whose sub-scores contribute to the final total score. The total score ranges from 0 to 88. A total score ≥ 33 indicates the probable presence of PTSD [39].

### Epigenetic analyses

Genomic DNA was extracted from blood samples using the QiaSymphony automated platform (Qiagen, Hilden, Germany).

Six-hundred nanograms of DNA were bisulphite-treated, to convert unmethylated cytosines into thymines, using the EZ DNA Methylation-Gold kit (Zymo Research, Irvine, CA, USA), according to the user manual. We analysed promoters/regulatory regions of 9 genes, already known to be associated with psychiatric disorders, as detailed in Table 1 [29, 40–48].

**Table 1:** Function and implication in human mental health of the selected genes

Gene (s)	Location brain	Function	Involvement in depression/PTSD	References
*ADCYAP1* (Pituitary Adenylate Cyclase-activating polypeptide)	Amygdala, hippocampus	Stimulates adenylate cyclase and increases cyclic adenosine monophosphate (cAMP) levels, resulting in the transcriptional activation of target genes The products of this gene are key mediators of neuroendocrine stress responses	Methylation changes associated with PTSD	Ressler et al. 2011 [46]
*BDNF* (Brain Derived Neutrophic Factor)	Prefrontal cortex	May play a role in the regulation of the stress response and in the biology of mood disorders	Increased promoter methylation associated with borderline personality disorder and PTSD	Kim et al. 2017 [43]
*CRHR1* (Corticotropin Releasing Hormone Receptor 1)	HPA axis	Encodes a G-protein coupled receptor that binds neuropeptides of the corticotropin releasing hormone family that are major regulators of the hypothalamic–pituitary–adrenal pathway	Peripheral hypo-responsive HPA-system and elevated CRH concentrations in cerebrospinal fluid in PTSD	Ströhle 2003 [48] Ding e Dai 2019 [42]
*DRD2* (Dopamine Type 2 Receptor)	Midbrain	Dopamine receptor D2, adenylate cyclase inhibiting, G protein coupled receptor superfamily, expressed in the basal ganglia, regulated by DNA methylation, involved in the control of appetite and growth hormone	Association with depression, anxiety and social dysfunction	Lawford 2006 [44]
*IGF2* (Insulin-Like Growth Factor 2)	Cerebral cortex	Encodes a member of the insulin family of polypeptide growth factors, which are involved in development and growth	Involved in PTSD, Schizophrenia, Bipolar disorder, Alcohol-related disorders, Alzheimer, memory impairment	Pardo 2019 [45]
*LSD1* (Lysine specific demethylase 1)	Frontal cortex	Acts as a co-repressor by mediating demethylation of H3K4me, a specific tag for epigenetic transcriptional activation	Protective of brain functionality in cortical and hippocampus. Changes in its expression are associated with neuropsychiatric disorders (e.g., depression and PTSD)	Cristopher et al. 2017 [41]
*NR3C1* (Nuclear Receptor Subfamily 3 Group C Member 1)	Prefrontal cortex	Encodes glucocorticoid receptor, which can function both as a transcription factor that binds to glucocorticoid response elements in the promoters of glucocorticoid responsive genes to activate their transcription, and as a regulator of other transcription factors	Increased activity or hyper-responsiveness of glucocorticoid receptor associated with PTSD	Chourbaji et al. 2008 [40] Ding e Dai 2019 [42]
*OXTR* (oxytocin and oxytocin receptor)	Brain cortex, cerebellum	The protein encoded by this gene belongs to the G-protein coupled receptor family and acts as a receptor for oxytocin. Its activity is mediated by G proteins which activate a phosphatidylinositol–calcium second messenger system	Involved in mood and social behavior regulation	Serati et al. 2021 [47]
*SLC6A4* *(Solute Carrier Family 6 Member 4)*	Amygdala	Encodes an integral membrane protein that transports the neurotransmitter serotonin from synaptic spaces into presynaptic neurons; Serotonin transporter whose primary function in the central nervous system involves the regulation of serotonergic signaling via transport of serotonin molecules from the synaptic cleft back into the pre-synaptic terminal for re-utilization	Involved in the etiology of mood and anxiety disorders	Park et al., 2019 [29]

*PTSD* post-traumatic stress disorder

Target genes were: *ADCYAP1, BDNF, CRHR1, DRD2, IGF2, LSD1/KDM1A, NR3C1, OXTR, SLC6A4.* Genomic regions to investigate were determined based on previously published data and in silico prediction of promoter sequences (FirstEF, http://rulai.cshl.org/tools/FirstEF/). For each locus, T7 5 'promoter-tagged PCR primers were designed using the EpiDesigner software (https://www.epidesigner.com, Agena Bioscience, San Diego, CA, USA). Primer sequences and targeted genomic regions are described in Table 2.

**Table 2:** Primer sequences and targeted genomic regions

Gene	Chromosome	Sequence ID	start—end	N° CpG sites	CpG coverage	PCR product size (bps)
*ADCYAP1*	18	AP000894.6	110,108—110,333	10	9	226
*BDNF*	11	NG_011794.1	4002—4,350	22	21	349
*CRHR1*	17	NG_009902.1	3971—4,263	24	21	291
*DRD2*	11	NG_008841.1	4581—4,789	12	12	209
*IGF2*	11	NG_008849.1	21,346—21,832	29	25	487
*LSD1/KDM1A*	1	NG_047129.1	5217—5,464	25	18	248
*NR3C1*	5	NG_009062.1	36,173—36,575	47	27	403
*OXTR*	3	KY798268.1	350—768	27	18	419
*SLC6A4*	17	NG_011747.2	4906—5,202	29	20	297

The methylation levels at the investigated loci were measured by the MassARRAY® EpiTYPER platform, with MassCleave settings (Agena Biosciences, San Diego, CA, USA), in accordance with manufacturer's recommendations and protocols, as previously described [49]. Mass spectra were acquired through a MassARRAY mass spectrometer (Agena Bioscience) and analyzed using the EpiTYPER^®^ MassARRAY^®^ software, which provided a quantification of methylated/unmethylated CpGs, with values ranging from 0 to 1.

### Statistical methods

Descriptive analyses were performed on the total sample. The two groups (cases and controls) were compared by independent sample *T* tests for quantitative variables (including the mean methylation levels of the selected genes) and *χ*^2^ tests for qualitative ones.

Statistical significance was set at *p* ≤ 0.05 and SPSS 26 version was used to perform the analyses.

## Results

### Analyses of clinical variables

A total of 68 healthcare professionals were included in the study. Descriptive analysis of the total sample and the comparison between cases and controls are reported in Table 3. 24.2% and 15.7% of the total sample presented clinically significant depressive and PTSD symptoms, reporting, respectively, a total score ≥ 10 and ≥ 33 at PHQ-9 and IES-r. As detailed in Table 3, controls showed more frequent history of anxiety/depression with respect to cases (*χ*^2^ = 5.72, *p* = 0.03). With regard to this latter aspect, only 4 subjects among the total sample reported past mood and anxiety symptoms (before the pandemic). On the contrary, cases versus controls presented higher PHQ-9 (*t* = 2.13, *p* = 0.04), PHQ-9 sleep item (*t* = 2.26, *p* = 0.03), IES-R total (*t* = 2.17, *p* = 0.03), IES-R intrusion (*t* = 2.46, *p* = 0.02), IES-R avoidance (*t* = 1.99, *p* = 0.05) mean total scores.

**Table 3:** Demographic and clinical variables of the total sample and of the two groups identified according to working in COVID or non-COVID wards

Variables		Total Sample *N* = 68	Non-COVID wards *N* = 29 (57.4%)	COVID wards *N* = 39 (42.6%)	*p* value
Age		38.06 (± 10.15)	39.10 (± 10.57)	37.28 (± 9.90)	0.47
BMI		23.33 (± 3.80)	22.97 (± 3.06)	23.60 (± 4.28)	0.51
Gender	Male	27 (39.7%)	14 (48.3%)	13 (33.3%)	0.32
	Female	41 (60.3%)	15 (51.7%)	26 (66.7%)	
Smoking status	Non-smoker	40 (58.8%)	17 (58.6%)	23 (59%)	0.78
	Past smoker	19 (28.0%)	9 (31.0%)	10 (25.6%)	
	Smoker	9 (13.2%)	3 (10.4%)	6 (15.4%)	
Number of cigarettes/day		1.62 (± 3.94)	1.83 (± 4.22)	1.46 (± 3.76)	0.71
Number of coffee cups/day		2.65 (± 1.83)	2.90 (± 1.86)	2.47 (± 1.81)	0.34
Number of alcohol units/month		13.88 (± 12.76)	16.93 (± 15.44)	11.69 (± 10.07)	0.10
Presence of hypercholesterolemia	No	58 (85.3%)	23 (79.3%)	35 (89.7%)	0.31
	Yes	10 (14.7%)	6 (20.7%)	4 (10.3%)	
Lifetime history of anxiety/depression	No	64 (94.1%)	25 (86.2%)	39 (100.0%)	**0.03**
	Yes	4 (5.9%)	4 (13.8%)	0 (0.0%)	
Presence of a sedentary lifestyle	Sedentary	14 (20.6%)	5 (17.2%)	9 (23.1%)	0.93
	Active (low intensity aerobic activities)	37 (54.4%)	16 (55.3%)	21 (53.8%)	
	Sporty	10 (14.7%)	5 (17.2%)	5 (12.8%)	
	Competitive sporty	7 (10.3%)	3 (10.3%)	4 (10.3%)	
Frequency of physical activity	Never	21 (30.9%)	11 (38.0%)	10 (25.6%)	0.38
	< 2 times in a week	21 (30.9%)	6 (20.7%)	15 (38.5%)	
	2–4 times in a week	23 (33.8%)	10 (34.4%)	13 (33.3%)	
	> 4 times in a week	3 (4.4%)	2 (6.9%)	1 (2.6%)	
Duration of a single session of physical activity (hours) missing *n* = 4	0	21 (32.8%)	11 (40.7%)	10 (27.1%)	0.35
	1	24 (37.5%)	11 (40.7%)	13 (35.1%)	
	2	17 (26.6%)	4 (14.9%)	13 (35.1%)	
	3	2 (3.1%)	1 (3.7%)	1 (2.7%)	
Frequency of using screen-based media	< 1 h/day	20 (29.4%)	7 (24.2%)	13 (33.3%)	0.53
	1–2 h/day	27 (39.7%)	11 (37.9%)	16 (41.1%)	
	> 2 h/day	21 (30.9%)	11 (37.9%)	10 (25.6%)	
History of COVID-19 missing *n* = 1	No	47 (69.1%)	21 (72.4%)	26 (66.7%)	0.79
	Yes	21 (30.9%)	8 (27.6%)	13 (33.3%)	
PHQ-9 mean scores		4.50 (± 3.94)	3.38 (± 3.41)	5.33 (± 4.14)	**0.04**
PHQ-9 sleep item mean scores		0.81 (± 0.83)	0.55 (± 0.69)	1.00 (± 0.89)	**0.03**
IES-R mean total scores		21.97 (± 20.58)	15.93 (± 21.53)	26.70 (± 18.75)	**0.03**
IES-R intrusion mean scores		8.45 (± 8.39)	5.69 (± 8.52)	10.62 (± 7.72)	**0.02**
IES-R avoidance mean scores		7.86 (± 7.92)	5.72 (± 7.62)	9.54 (± 7.84)	**0.05**
IES-R hyperarousal mean scores		5.74 (± 5.88)	4.55 (± 6.23)	6.68 (± 5.49)	0.15

Standard deviations for quantitative variables and percentages for qualitative variables are reported into brackets

In bold statistically significant *p* resulting from *χ*^2^ or unpaired student’s *t* tests ≤ 0.05

*BMI* body mass index, *IES-R* impact of event scale-revised, *PHQ-9* patient health questionnaire-9

### Methylation analyses

**Fig. 1:**
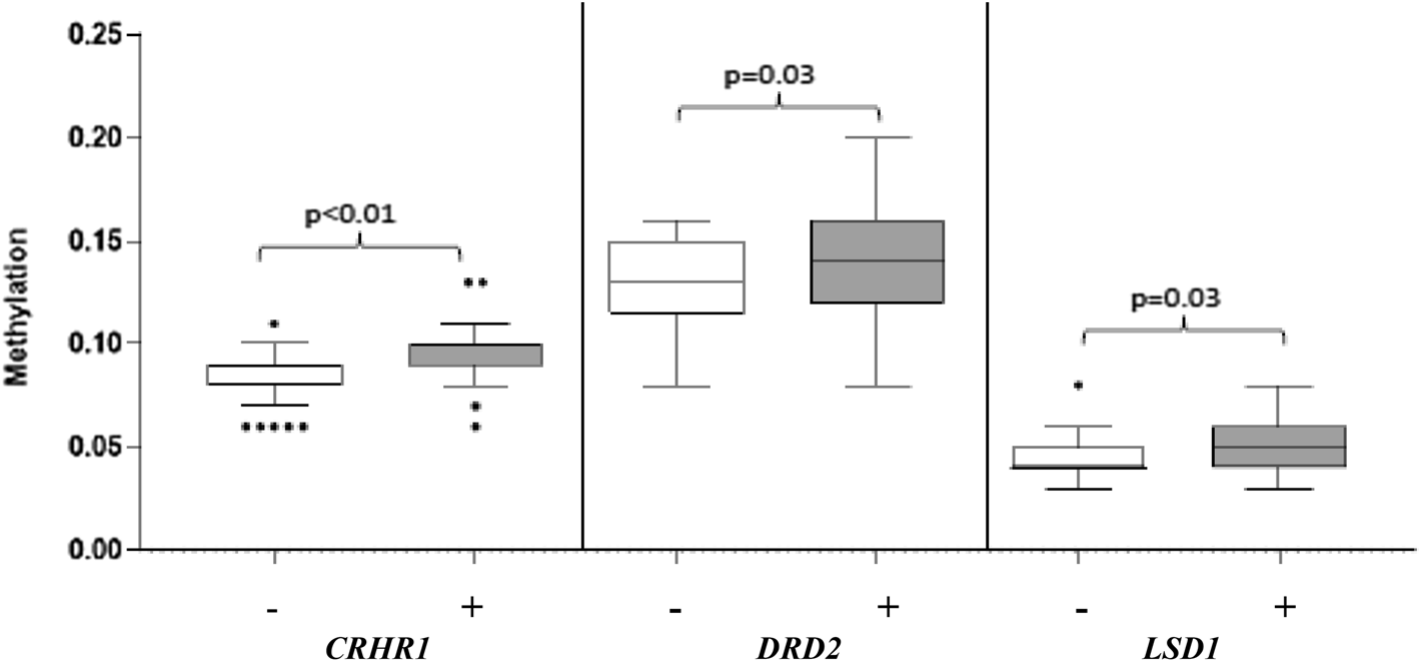
Box-plots representing minimum, first quartile, median, third quartile, and maximum methylation values of genes resulted significantly hypermethylated in cases ( +) versus controls (−)

Methylation levels of 3 promoter regions, *CRHR1*, *DRD2*, *LSD1* were significantly higher in cases versus controls. Indeed, as detailed in Table 4 and depicted in Fig. 1, mean methylation levels (± standard deviation) of cases versus controls were: 0.092 (± 0.01) versus 0.084 (± 0.01) (*p* < 0.01) for *CRHR1;* 0.143 (± 0.03) versus 0.128 (± 0.02) (*p* = 0.03) for *DRD2*; 0.050 (± 0.01) versus 0.044 (± 0.015) (*p* = 0.03) for *LSD1,* respectively. No significant differences were found in the other investigated genes (Table 4).

**Table 4:** Methylation values of all investigated regions

Gene	Methylation in controls					Methylation in cases					*p*
	Mean	SD	Median	Min	Max	Mean	SD	Median	Min	Max	
*ADCYAP1*	0.140	0.044	0.128	0.093	0.313	0.141	0.043	0.133	0.095	0.320	0.89475
*BDNF*	0.095	0.035	0.090	0.044	0.212	0.091	0.018	0.089	0.039	0.128	0.51807
*CRHR1*	0.084	0.013	0.085	0.061	0.109	0.092	0.010	0.091	0.061	0.128	**0.00420**
*DRD2*	0.128	0.023	0.127	0.083	0.161	0.143	0.028	0.141	0.081	0.204	**0.02718**
*IGF2*	0.497	0.059	0.486	0.343	0.612	0.501	0.060	0.494	0.369	0.705	0,78,694
*LSD1*	0.044	0.010	0.042	0.029	0.076	0.050	0.011	0.048	0.028	0.077	**0.03333**
*NR3C1*	0.047	0.015	0.045	0.028	0.090	0.047	0.013	0.045	0.021	0.087	0.93112
*SLC6A4*	0.051	0.009	0.048	0.037	0.082	0.053	0.008	0.053	0.038	0.073	0.25221
*OXTR1*	0.247	0.038	0.249	0.179	0.315	0.257	0.032	0.255	0.161	0.334	0.27992

*p *values of genes showing significant differences between cases and controls are highlighted in bold

*Min* minimum, *Max* maximum, *SD* standard deviation

Moreover, in cases stratified according to gender, higher methylation levels of *OXTR* were found in females (0.26 ± 0.03) versus males (0.24 ± 0.03) (*p* = 0.05).

## Discussion

A large number of subjects included in our sample reported clinically significant depressive and PTSD symptoms with frequencies higher than those reported in general population [6]. Considering the two groups, identified according to the type of work, frontline health professionals resulted to be more affected by the pandemic as showed by the higher mean total scores at psychometric scales with respect to non-frontline workers. The findings are in agreement with those reported by the available literature indicating a negative effect of pandemic on mental health of most people [50] and in particular in frontline health professionals [6, 11]. It is not surprising that in our sample non-frontline workers presented more frequently a history of anxiety or depression (before the pandemic) than frontline ones, because they were likely to be excluded from more extenuating jobs in the light of their vulnerability to stress and consequently to clinically significant depressive and PTSD symptoms. However, this aspect even supports more the deleterious effect of frontline work on mental well-being of health workers as these subjects were basically less vulnerable to the development of psychiatric disorders than non-frontline professionals. Similar results were reported in a study conducted in Barcelona, where a significant number of frontline healthcare workers suffered from significant depression and anxiety during the pandemic, but few of them showed a history of anxiety and depression before COVID-19 outbreak [51].

With regard to epigenetic results, frontline workers (versus non-frontline ones) showed a higher degree of methylation of genes regulating HPA axis (*CRHR1)* or dopamine neurotransmission (*DRD2* and *LSD1*). Available literature indicates that HPA axis is frequently involved in stress responses including the onset of cognitive or mood symptoms [52]. In agreement with our data, a recent study reported that during COVID-19 pandemic first-line health professionals, particularly physicians, presented higher hair cortisol concentrations with respect to workers not in direct contact with patients [14]. On the other hand, epigenetic modulation of genes involved in dopamine transmission may account for the development of behavioural and substance addictions, anhedonia and depressed mood, or psychotic symptoms. Of note, *DRD2* promoter methylation was reported to be directly associated with the severity of alcohol misuse [53]. Stress is a well-known risk factor for alcohol misuse [54] and, although this aspect did not emerge in our sample, some authors reported an increase in alcohol use in health professionals assisting patients with COVID-19 [55]. According to our results, *DRD2* would result less expressed in cases versus controls. This aspect is in agreement with results from animal models showing that *DRD2* mRNA expression is reduced in case of chronic mild stress and in case of expression of depressive-like behaviours [56]. Furthermore, a lower expression of *DRD2* (e.g., for the presence of Taq1 polymorphism) was reported to be associated with the onset of PTSD symptoms [57], and in our sample *DRD2* resulted to have a higher degree of methylation in cases who in turn presented more severe PTSD symptoms than controls.

Finally, regarding the different methylation of *OXTR* in female cases than male ones, this is not surprising, because oxytocin is a neuropeptide that modulates the behavioural response to stress and has a different effect according to gender. While in males oxytocin facilitates the change of anxiety (in response to stress) in happiness, in females this neuropeptide favours relaxation [31].

It should be noted that the reported changes in DNA methylation levels are subtle (generally < 5%), and this is likely due to the fact that higher differences in methylation would not be tolerated by cells, reinforcing the role of epigenetic regulation in modulating the expression of the investigated gene, in one hand, and their role in psychiatric disorders, on the other hand.

Another issue, that would deserve perspective studies, is whether epigenetic changes observed in frontline health workers will be stable over time or sensitive to external interventions.

The study limitations include:

(1) pharmacological treatment for medical diseases (e.g., for dyslipidaemia) might have influenced some of our epigenetic results, although no statistically significant clinical differences (a part from the history of anxiety and depression) were detected between cases and controls;

(2) the recruitment in a single centre in a European country that limits the generalization of the present findings (with regard to this point, it is useful to highlight that Italy was the first European country to face the emergency of pandemic and it is one of the European countries with the most deaths due to the COVID-19 pandemic);

(3) the clinical information acquired by subjects might not be always accurate, although this limitation is mitigated by the fact that sample consisted of health professionals;

(4) the analysis of DNA methylation of peripheral blood lymphocytes (PBLs) instead of brain cells. Nevertheless, we assumed that methylation levels in PBLs could reflect those of the brain cells, based on previous evidences. Indeed, it has been demonstrated that DNA methylation levels at PBLs mirror those of brain cells [30, 31], at that for this reason PBLs can be used as reliable peripheral markers of psychiatric conditions, such as major depressive disorder [32, 33]. Recently, the finding that blood–brain barrier permeability is increased in individuals with central nervous system diseases reinforces the idea that it is possible to retrieve in peripheral blood molecules originating from brain and this, in turn, facilitates the diagnosis of psychiatric/neurological disorders by the analysis of PBLs [34].

The results of our study show that frontline health workers were more negatively affected by the pandemic than healthcare professionals not in direct contact with patients in the case of a single center in Italy (a single European Country). Future multi-centric studies with larger samples and investigating different biological systems are necessary to support the preliminary results of the present study and to have a more complete picture of the effects of acute stressful events (such as a pandemic) on mental health.
